# Molecular dynamics simulations of positively selected codons in FcγRI reveal novel biochemical binding properties

**DOI:** 10.1002/2211-5463.70247

**Published:** 2026-05-01

**Authors:** David A. Young, Omar Arias‐Gaguancela, Fiona Gibson, Morgan Grainger, Jodie Score, Amit Nathubhai, Mauricio Cafiero, Lee Richard Machado

**Affiliations:** ^1^ Molecular Bioscience Research Group, Centre for Physical Activity and Life Sciences University of Northampton UK; ^2^ SciLearningWorkshops Dallas TX USA; ^3^ School of Chemistry, Food and Pharmacy University of Reading Reading UK

**Keywords:** codon‐based selection tests, FcγRI, molecular dynamics, molecular geometry, phylogeny

## Abstract

FcγRI is a high‐affinity receptor for IgG, associated with autoimmune disease pathology and determines clinical responses to antibody‐based immunotherapies. FcγRI has a complex evolutionary history that is not fully understood, and to address this we explored signatures of positive selection in the receptor's functional gene, *FCGR1A,* using codon‐based selection tests on aligned 1–1 orthologous sequences from placental mammals (*n* = 32). Signatures of positive selection have occurred at several locations within the gene, with two sites (H^148^ (M2a ω 0.997 & M8 ω = 0.993)) and (W^149^ (M2a ω = 0.999 & M8 ω = 1.000)) exhibiting highest posterior probabilities, suggesting strong evidence of positive selection; these positions are known to form one of the FcγRI‐IgG binding interfaces. We employed ancestral reconstruction to statistically infer prior codon sequences at these sites and identified ancestral H^148^P and W^149^R codons at different nodes in the phylogeny. Employing molecular dynamics simulations, we determined how evolutionary changes at these sites may have influenced the binding of FcγRI‐IgG of modern‐day *Homo sapiens*. Measuring RMSD, free energy, radius of gyration, hydrogen bond formation, and analyzing free energy landscapes, we demonstrate that structural instability between mutant structures vs the WT counterpart; however, overall binding potential increases at position 148, yet decreases at 149 in potential. H^148^P protonation at physiological pH remains similar, yet during acidotic calculations, protonation is likely reduced, with predicted reduction in affinity for IgG. While ancestral W^149^R substitutions demonstrate an implication for electron conjugation. Examining key sites at this binding FcγRI‐IgG interface, our data demonstrate that these two codons have evolved in humans to be relatively insensitive to shifts in pH promoting a more stable interaction with the Fc portion of IgG during diseases that promote acidosis.

## Graphical abstract

Evolutionary analysis across 32 placental mammals identified positive selection at residues H148 and W149 in the immune receptor FcγR1. Ancestral reconstruction combined with molecular dynamics simulations reveals how these mutations may influence receptor structure and dynamics, providing insight into the evolution of antibody recognition and immune function.

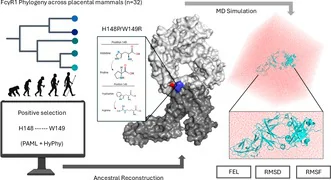

**Abbreviations**
Å: Ångström
atm: atmosphere (pressure unit)
BAR: Bennett acceptance ratio
BeB: Bayes Empirical Bayes
BUSTED: Branch‐site Unrestricted Statistical Test for Episodic Diversification
Ca^2+^
: calcium ion
dN: nonsynonymous substitution rate
dS: synonymous substitution rate
FCGR1A: Fc gamma receptor 1A gene
FcγR: Fc gamma receptor
FcγRI: fragment crystallizable gamma receptor I
FEP: Free Energy Perturbation
FUBAR: Fast Unconstrained Bayesian AppRoximation
GARD: Genetic Algorithm for Recombination Detection
GPI: glycosylphosphatidylinositol
IgG: immunoglobulin G
IP3: inositol triphosphate
ITAM: Immunoreceptor Tyrosine‐based Activation Motif
ITIM: Immunoreceptor Tyrosine‐based Inhibitory Motif
K: kelvin
kcal/mol: kilocalories per mole
kJ/mol: kilojoules per mole
LRT: likelihood ratio test
MD: molecular dynamics
MEME: Mixed Effects Model of Evolution
ML: maximum likelihood
MM/GBMV: Molecular Mechanics/Generalized Born Molecular Volume
MM/GBSA: Molecular Mechanics/Generalized Born Surface Area
NCBI: National Center for Biotechnology Information
NPT: Constant Number of particles, Pressure, Temperature ensemble
ns: nanoseconds
NVT: Constant Number of particles, Volume, Temperature ensemble
PAML: Phylogenetic Analysis by Maximum Likelihood
PBC: Periodic Boundary Conditions
PDB: Protein Data Bank
PKC: protein kinase C
PLCγ: phospholipase C gamma
PME: Particle Mesh Ewald
ps: picoseconds
Rg: radius of gyration
RMSD: root mean square deviation
RMSF: root mean square fluctuation
SNP: single nucleotide polymorphism
TIP3P: Transferable Intermolecular Potential with 3 Points (water model)
UFBoot: ultrafast bootstrap
WT: wild‐type
ΔG: change in Gibbs free energy
ω (d_N_/d_S_): ratio of nonsynonymous to synonymous substitutions

Fragment crystallizable gamma receptor 1 (FcγRI) is associated with several autoimmune diseases including Kawasaki disease and systemic lupus [1]. Additionally, FcγRI, also referred to as CD64, shows expression that is markedly upregulated on neutrophils and monocytes in sepsis patients [2]. The additional FcγR family members are important in mediating antibody immunotherapies. FcγRI is ubiquitously expressed on both monocytes and polarized macrophages and binds directly to the heterodimeric Fc portion of immunoglobulin gamma (IgG), most often the IgG_3_ and IgG_1_ isotypes at two major positions [3]. The first binding site, FcγRI H^148^, binds to G^236/237^ and L^238^ of the Cγ2‐B chain of IgG. The second site, the ‘^173^KHR^175’^ motif of FcγRI, binds to the Cγ2‐A chain of IgG [4]. IgG is the most abundant free circulating Ig in serum and has a longer half‐life (approximately 28 days) compared to other Ig subtypes, such as IgA (approx. 5 days) [5, 6] This increased ligand half‐life, combined with inducible expression of FcγRI on additional innate cells, triggers multiple downstream signaling pathways, including phospholipase‐Cγ (PLCγ) pathway, inositol triphosphate (IP3), and protein kinase‐C (PKC) activation, ultimately leading to intracellular Ca^2+^ flux and cellular effector function [7].

FcγRI is the only high affinity receptor found in the FcγR family with nanomolar (nM) binding affinity, while the low‐affinity receptors bind in the micromolar (μM) range [1]. FcγRI is structurally unique, with three extracellular domains in contrast to the two domains of the low‐affinity receptors [8]. FcγRI, FcγRIIa, and FcγRIIc have immunoreceptor activator motifs (ITAM), and only FcγRIIb has an immunoreceptor inhibitory motif (ITIM). FcγRIIIa has a common activator γ‐chain while FcγRIIIb is glycosylphosphatidylinositol (GPI) linked with no intracellular chain [9]. The activation and inhibitory signals of the FcγR family form an activation/inhibitory ratio (A/I ratio) that allows a finely tuned immune response upon receptor–ligand binding, eliciting a downstream response [2].

FcγRI is not easy to examine *in vitro* and often cross‐links monomeric IgG, saturating the receptor [10]. Yet, the ^173^KHR^175^ motif has recently been characterized by Lu *et al*. (2023), who showed that the positively charged residues within this motif form electrostatic and polar contacts with residues L^173^ and N^265^ of IgG Cγ2‐A chain, which is considered key to the receptor's high affinity status. Conserved domains are a common occurrence across the receptor family, where low‐affinity receptors also maintain the L^120^ to N^265^ link but via hydrogen bond formation [4, 8], in contrast to the bridge formation in FcγRI. Although somewhat conserved at position 120, when mutations do occur at L^120^ for the high‐affinity receptor, L^173^ acts as a salt‐proxy substitute contact point. FcγRI L^173^ forms a hydrophobic interaction allowing H^172^ and N^175^ of FcγRI to extend into an opening within the Fc portion of IgG, toward the glycan core and reach an interaction point on the distal carbohydrate of IgG N^297^. This allows FcγRI R^175^ to connect with the side chain of the adjacent H^174^, protonating the C4‐hydroxyl group of the mannose. Protonation of the mannose C4‐hydroxyl likely keeps FcγRI R^175^ 1.5–2.5 Å from the third hydroxyl of the proximal N–Acetyl–glucosamine [4]. The importance of the highly conserved N^297^ residue found beneath the hinge region of the CH_2_ domain on IgG_1_ is well‐established for interactions with the low‐affinity receptors and considered equal in the high affinity receptor but not fully examined to date.

There remains uncertainty surrounding the interaction of FcγRI^H148/W149^‐IgG^Cγ2‐B^, with the amphiprotic H^148^ allowing multiple binding partners based on proximity; this is reliant on the position of W^149^, which presents mild fluctuations in binding affinity. To further complicate interpretation, IgG‐^FAB^ antigen binding also appears to exhibit differentiated conformational changes depending on the antigen, causing IgG‐^CH3/CH2^ relative or dihedral angle rotations [11], allowing zinc ion interactions at H^174^ of FcγRI. These all contribute to potential intracellular autophosphorylation and activation of the receptors' intracellular ITAM‐bearing domain, differentiating biochemical pathway activation for immunological responses [12]. However, there is evidence of SNP variation at V^391^ as described by Brandsma *et al*. [13], which they determine a regression in intracellular calcium mobilization.

The FcγRI functional gene, *FCGR1A* (*Chr 1q21.2*) (GRCh38/hg38), is thought to have been subject to evolutionary inversion and duplication events leading to the rise of two additional pseudogenes *FCGR1B* (Chr 1p11.2) and *FCGR1C* (Chr 1q21.1) forming a pericentromeric loci [14]. The pseudogenes, however, exert minimal to no expression in the vast majority of populations, making *FCGR1A* the primary gene of interest. Furthermore, the detail of mutation and evolution of the high affinity genes remains poorly defined compared to the low‐affinity loci (1q23.3) [15, 16].

Here, we explore the functional gene, *FCGR1A* phylogeny, and establish signatures of positive selection at the gene level and site‐specific locations across placental mammals and address the possibility that rapid evolution may have impacted receptor–ligand binding. By using 100 ns molecular dynamics simulations, we evaluated binding properties such as total energy, hydrogen bond formation, radius of gyration, free energy landscapes, and root mean square deviation/fluctuations changes between IgG and modern day FcγRI compared to ancestral FcγRI substitution variants. In addition to testing ancestral substitutions, alanine mutants were also evaluated *in silico*, exploring the contribution of sidechain activity to receptor binding.

## Methods

### Sequence alignment, phylogeny and ancestral reconstruction

Putative 1–1 orthologous sequences of *FCGR1A* (NM_000566.3/ GenBank:X14355.1) were identified via Gene Orthology prediction methods implemented in the Ensembl genome browser and through the National Center for Biotechnology Information (NCBI) nucleotide database (*n* = 32). Sequences were aligned with the MEGA7 ClustalW function, manually inspected, and initiating methionine's, scaffolds, and premature stop codons subsequently removed, allowing generation of an unrooted tree using maximum likelihood with DNA Maximum Likelihood (DNAML) through the PHYLIP 3.6 package. Analysis employed a Kimura 2‐parameter model with gamma distribution (K2‐G), and bootstrap analysis with 1000 replicates. For robustness, the alignment file was then imported into W‐IQ‐TREE where a phylogenetic tree was constructed by maximum likelihood (ML). The substitution model was selected using ModelFinder implemented within the IQ‐TREE software, and node support was assessed via ultrafast bootstrap approximation (UFBoot) *n* = 1000 replicates (Trifinopoulos *et al*., 2016), both yielding the same tree.

Ancestral sequence reconstruction was performed using PAMLx (Phylogenetic Analysis by Maximum Likelihood) to infer the most likely ancestral sequences at internal nodes of the phylogenetic tree. The analysis was conducted under the maximum likelihood framework, providing estimates of ancestral states based on the placental mammal orthologous sequences and their phylogeny. The phylogenetic tree represented evolutionary relationships among the taxa and was inserted into the PAMLx interface; the program was used to estimate ancestral sequences at each internal node using an M0 model. The ancestral reconstructions were carried out using the ‘ancestral’ mode, which estimates the probability of each possible ancestral base aligned as codons for each position in the sequence alignment via PAMLx. These reconstructions were visualized in MEGA7 to identify conserved and divergent regions across the evolutionary tree. The results were further analyzed to identify potential functional or evolutionary changes in the ancestral sequences.

### Detection of recombination

Recombination events were assessed using the GARD (Genetic Algorithm of Recombination Detection) method implemented in HyPhy [17], which detected no significant evidence of recombination in the dataset [18].

### Gene‐wide and site‐specific positive selection tests

Codons were analyzed through phylogenetic analysis of maximum likelihood to infer positive selection across species. Relationships between species were determined and observed for evidence of positive selection via PAML (v4.10) [19, 20]. Site models M1a vs M2a and M7 vs M8 were compared. These models test whether positive selection, as defined by ω > 1, has acted on a gene. M1a is the model assuming a null hypothesis that all codons fall into two classes: (1) those that evolve neutrally (ω = 1) or (2) those that evolve with purifying selection (0 < ω <1). M2a allows codons to fall into another class: those showing positive selection (ω > 1).

Therefore, by comparing the log likelihood of the data‐fitting model M1a with the log likelihood of the data‐fitting model M2a, we obtain a significance level testing the hypothesis that certain codons are under positive selection. Positive selection defined by ω > 1, suggests positive selection acted on a gene. Similarly, M7 models ω values with a beta distribution constrained between 0 and 1, whereas M8 adds an additional class permitting ω > 1.

### Confirmation of positive selection using hypothesis testing using phylogenies (HyPhy)

To further investigate signatures of positive selection, several evolutionary HyPhy packages were applied. BUSTED (Branch‐Site Unrestricted Statistical Test for Episodic Diversification) [21] was used to determine if positive selection had occurred across species at a whole gene‐level (nonsite‐specific). MEME [22] was employed to determine if positive selection had occurred at site‐specific levels under a proportion of branches. FUBAR [23] assumes equal pressure across the phylogeny but uses the Bayesian approach for d_N_ vs d_S_ ratio inference.

### Molecular dynamic simulations

PyMol (V3.0 Schrodinger, LLC) was used to map codons under the strongest evidence of selection to a published crystal structure (PDB_4W4O). Ancestral counterparts were generated via the mutagenesis function following receptor–ligand sequence determination. In addition to substituting ancestral codons, alanine substitutions were introduced at PDB_4W4O author positions 148 and 149, providing mutants that maintained structural integrity while removing the side chains of histidine and tryptophan respectively *n* = 7 (6 mutants & 1 wild‐type). Before simulation, additional water molecules were manually removed that inferred steric clashes, and the receptor parameterized at positions 148 and 149, inspected with additional neutral Ɛ & δ protonation inspected for tautomeric histidine.

To characterize the molecular interface between the Fc region of IgG and FcγRI, we employed PatchDoc (v1.3) and FireDoc (v1.1) on PDB_4W4O, focusing on whether residues 148 and 149 contribute additional binding interactions beyond the canonical Fc–FcγRI interface using a 5 Å cutoff. Building on these findings, we applied the Computed Atlas of Surface Topography of Proteins, CASTp‐Fold (v3.0), to map and quantify alterations in binding pocket architecture, with particular attention to changes in pocket volumes (Å^3^).

For energy minimization, the Avogadro interface (v1.2) [24] was employed, with a maximum force threshold of 10.0 kJ·mol^−1^·nm^−1^ and conjugate gradient minimization (50 000 steps). After steepest descent was determined to remove clashes, subsequent strain reduction was employed in GROMACS (vLGPL‐2.1) to refine geometry, applying an AMBER_99 force field to establish optimized parameters with adjusted box pressures to satisfy NVT/NPT restraints [24].

To transition from GROMACS (vLGPL‐2.1) to Desmond (v22.1 Schrödinger) coordinates and box vectors were exported and parameterization was aligned to match simulation physics slowly for equilibration.

Desmond (v22.1 Schrödinger) was then used for simulation (1× replica, 100‐ns duration) with an initial temperature of 310 K maintained by a Nosé–Hoover chain thermostat, and constant pressure of 1 atm enforced by the Martyna–Tobias–Klein barostat. The forcefield was ‘OPLS3’. Periodic Boundary Conditions (PBC) were applied with a 10 Å buffer around the solute. The integration timestep parameters were set at 2 fs with M‐SHAKE constraints applied to hydrogen bond lengths. A TIP3P solvation model was used with NaCl set to 0.15 m to represent the aqueous environment. Long‐range electrostatics were calculated using particle mesh Ewald (PME) with a real‐space cutoff of 9.0 Å. Free energy calculations employed the default Bennett Acceptance Ratio (BAR) λ windows parameters within the FEP+ module, while the Molecular Mechanics‐Generalized Born Volume Integral (MM/GBVI) method was used to estimate relative binding energies. Root Mean Square Deviation (RMSD) for each calculated Cα atom was aligned and recorded at 1‐ps intervals. Validation of transition was ensured through manually inspecting energy spikes, temperature & pressure stability and sudden RMSD movement, allowing for identification of parameter changes. RMSD (Figs S1 and S2) and radius of gyration (Rg) metrics (Figs S3 and S4) also determined trends of plateau, a common practice for 100‐ns simulations of one replicate [25, 26].

## Results

### *FCGR1A* orthologues have experienced a complex evolutionary history in placental mammals and exhibit signals of positive selection at one of the two known IgG binding sites

**Fig. 1:**
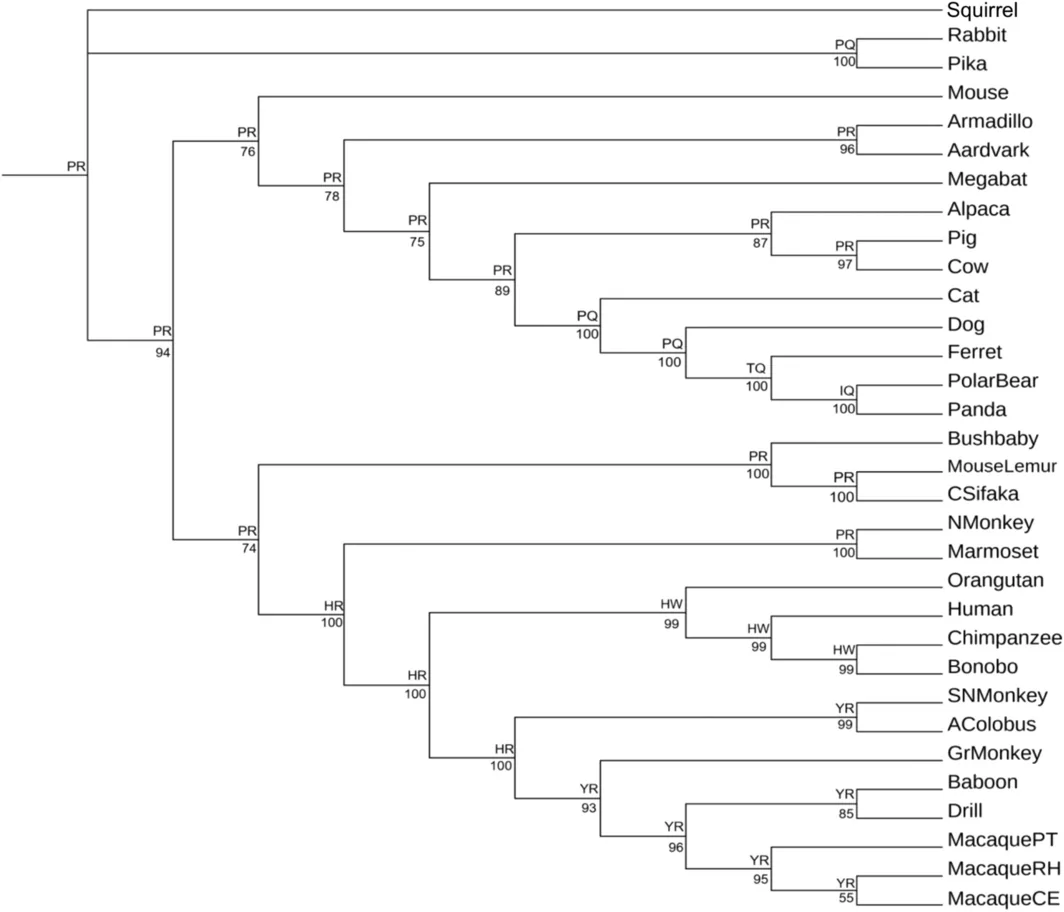
PAML Bootstrap (*n* = 1000 replicates) used to create a maximum‐likelihood (unrooted) tree (W‐IQ‐TREE) of the FCGR1A coding sequences across placental mammals. Numbers represent percentage bootstrap support. Letters represent the ancestral codon at lineage divergence.

**Fig. 2:**
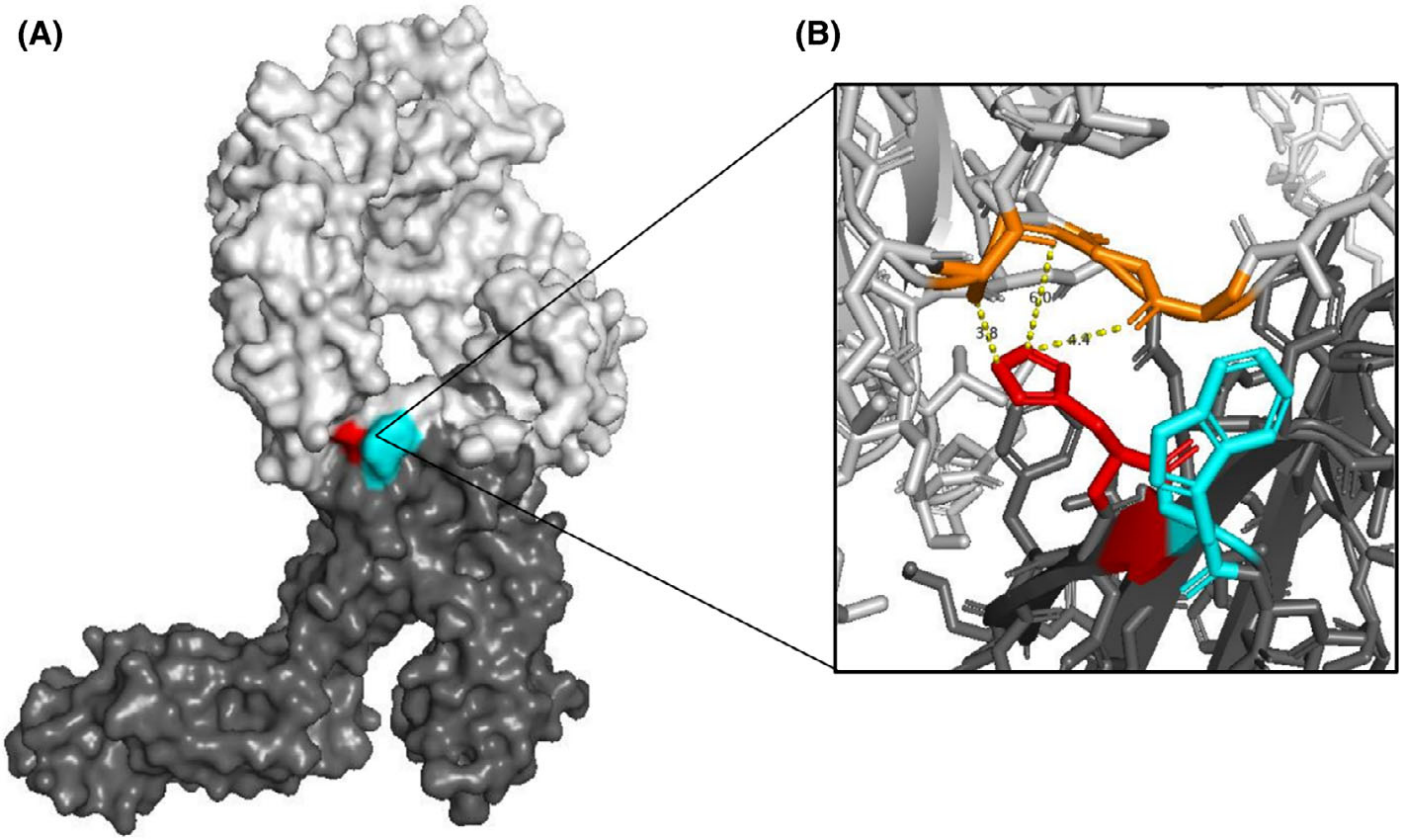
(A) FcγRI (Gray) in complex with the Fc portion of IgG (white) obtained from PDB and visualized with PyMOL, with the two strongest residues under positive selection. (B) Chemical composition of position 148, histidine (red) and position 149, tryptophan (cyan), and their interaction with Glycine 235 & 236 of IgG (copper) and their distance between each other in Å.

Sequences from *FCGR1A* orthologues of 32 placental mammalian species were aligned for evolutionary analysis and an unrooted maximum‐likelihood phylogenetic gene tree was constructed to infer the relationship among the different taxa, which was supported by bootstrap analysis (Fig. 1). To test whether the *FCGR1A* sequences contained sites with a substitution rate ratio (ω or d_N_/d_S_) larger than 1, a likelihood ratio test was used in PAML to compare two models, either M1a vs M2a or M7 Vs M8 and using either the derived gene tree or species trees (Table 1). The constrained models (M1a and M7) were rejected in favor of the model allowing ω > 1 (M2a and M8), concluding that at least one site in the sequence has evolved under positive selection. As this test indicated statistical evidence for the presence of a proportion of sites evolving under positive selection, we asked whether specific sites evolved by calculating the posterior probability that ω > 1 for each site. Bayes empirical Bayes (BeB) tests were then used to identify positively selected sites. Ten codons had a posterior probability of >0.90 indicating positive selection acted at those sites (Table 2). Positions 148 and 149 had the strongest evidence of positive selection with a posterior probability >0.99 (Table 2 boxed section). These two sites were then mapped to the published crystal structure (PDB_4W4O) of human FcγRI both of which are located at a binding interface with IgG (Fig. 2 (gray)).

**Table 1:** Multiple species alignment measured models of best fit M1a (neutral model) vs M2a (positive model) and M7 (neutral/negative model) vs M8 (positive model) (cutoff set to *X* ^2^ 9.21 (*P* = 0.01)), using species and gene tree phylogenies, with the log likelihood and adjusted *P* values for fit determined.

Model No.	Tree	Parameters		Log L'hood	*P* value
M1a vs M2a	Species	M1a = 0 < ω0 < 1, ω1 = 1	M2a = 0 < ω0 < 1, ω1 = 1, ω2 ≥ 1	29.6702	3.607 × 10^−7^
M7 vs M8	Species	M7 = p, q	M8 = p, q, ω2 ≥ 1	44.5507	2.118 × 10^−10^
M1a vs M2a	Gene	M1a = 0 < ω0 < 1, ω1 = 1	M2a = 0 < ω0 < 1, ω1 = 1, ω2 ≥ 1	31.956	1.15 × 10^−7^
M7 vs M8	Gene	M7 = p, q	M8 = p, q, ω2 ≥ 1	46.689	7.271 × 10^−11^

**Table 2:** Codons under strongest evidence of selection (mapped to the published crystal structure), omega values for M2a and M8 models. ** = to strongest evidence of selection.

Position in FcγRI	Tertiary position in FcγRI	Amino acid	Posterior probability site ω > 1% model M8	Posterior probability ω > 1% model M2a
42	Extracellular D3	H	0.974*	0.817
48	Extracellular D3	L	0.951*	0.629
148	Extracellular D2	H	0.997**	0.993**
149	Extracellular D2	W	0.999**	1.000**
236	Extracellular D1	L	0.980*	0.566
256	Extracellular D1	G	0.985*	0.92
313	Intracellular domain	I	0.962*	0.614
331	Intracellular domain	S	0.980*	0.824
338	Intracellular domain	K	0.985*	0.891
344	Intracellular domain	L	0.957*	0.583

To ensure our findings were not confounded by recombination events, we performed a GARD analysis to screen alignments for potential recombination breakpoints. GARD tested 2412 models across 724 potential breakpoints and detected no evidence of recombination. The analysis yielded a Δc‐AIC of 2.11 × 10^4^ compared to the null model and 2.29 × 10^−4^, compared to the single‐tree multiple‐partition model indicating the identified breakpoints did not represent true topological incongruence.

Employing the BUSTED framework (Murrell *et al*. [21]), we performed a gene‐wide test to evaluate whether sites 148 and 149 experienced episodic positive selection across the species tree. (Table 3). Optimized ω values were 1.007 and 0.855 for sites 148 and 149, respectively, suggesting these sites underwent positive selection on at least one branch of the tree. The log‐likelihood scores of −62.624 for position 148 and −73.134 for position 149, along with their site‐specific log‐likelihood contributions (0.015 for position 148 and − 0.314 for position 149), further support this conclusion. Additionally, BeB factors of 10 for position 148 and 15 for position 149 also indicate a moderate to strong signal of episodic selection.

**Table 3:** Unrestricted statistical test via BUSTED. ω > 1 for constrained (column 2) and optimized models (column 3), log‐likelihood score (column 4), E_post_ [α] (Empirical Posterior Expectation (column 5)) and likelihood ratio score values (column 6).

Position in FcγRI	ER ω > 1 constrained	ER ω > 1 opti	LogL	Epost[α]	LR
148	4.175	1.007	−62.624	1.493	0.015
149	4.339	0.855	−73.134	1.493	−0.314

MEME analysis was used to determine whether the individual sites 148 and 149 have undergone episodic or pervasive positive selection by using a mixed effect maximum likelihood framework (Table 4). MEME estimated evolutionary rates (ω) by calculating a single synonymous substitution rate (α) and two nonsynonymous substitution rates (β− and β+), corresponding to neutral/purifying evolution and potential positive selection, respectively. Sites 148 and 149 were confirmed as being under episodic positive selection, with significantly higher β+/α ratios compared to the null model, which disallows positive selection. The likelihood ratio test (LRT) confirmed that the alternative model, which permits positive selection, better explained the data at these sites. This suggests that at certain evolutionary branches, Sites 148 and 149 have experienced adaptive changes, likely reflecting functional or structural importance.

**Table 4:** Residues under strongest evidence of positive selection based on a mixed effects model. The α column defines synonymous substitution rates at each site, β + defines the nonsynonymous substitution rate of positive selection. LRT is the likelihood ratio test statistic for episodic diversification, Asymptotic *P* value for episodic diversification. Followed by branch numbers under selection and their lengths.

Position in FcγRI	α	β +	*P*+	LRT	*P*‐value	Branches under selection	Total branch length
148	0.38	8.01	0.45	8.21	0.01	2	2.78
149	0.8	4.52	1	6.01	0.02	4	3.45

FUBAR analysis further identified selection at positions 148 and 149 as being under selection using a Bayesian approach to infer (d_N_) and (d_S_) substitution rates on a per‐site basis. FUBAR assumes the selection pressure for each site is constant along the entire phylogeny (Table 5). Among several sites under positive selection, positions 148 and 149 were again under the strongest evidence of selection. The synonymous substitution rates (α) were estimated at 0.933 and 0.937, respectively, while the nonsynonymous substitution rates (β) were 3.211 for position 148 and 6.88 for position 149. The differences between β and α (β – α) were 2.278 and 5.942, suggesting an excess of non‐synonymous substitutions, characteristic of selective pressure. The probabilities that α < β (indicating selection) were high for both sites: 0.951 for position 148 and 0.98 for position 149. Bayesian factors further reinforced this, with values of 33.512 for site 148 and 87.727 for site 149; in addition, the *P*+ value for position 149 is = 1, which means all branches evidence signatures of selection, providing strong evidence of positive selection. This is further observed with the α‐β values of 5.942 at position 149 and 2.278 at position 148, signifying the previous ω values are directional toward nonsynonymous substitution.

**Table 5:** Identifies the positions in FcγRI that are under selection. Column α defines synonymous substitution rates at each site, β + defines the non‐synonymous substitution rate of positive selection; furthermore, there are probability factors and Bayesian factor.

Position in FcγRI	α	β	β – α	Prob [α > β]	Prob [α < β]	Bayes factor [α < β]
149	0.937	6.88	5.942	0	0.98	87.727
148	0.933	3.211	2.278	0	0.951	33.512

### Ancestral reconstruction

The interface of FcγRI is critical for receptor–ligand binding. To explore the evolutionary changes at this interface, we reconstructed ancestral sequences using the *FCGR1A* phylogeny, focusing on codons at Sites 148 and 149 (Fig. 1). The ancestral codon at position 148 was identified as ‘CCC’, which encodes proline. This codon was conserved from the common ancestor shared with Hominidae (great apes) approximately 60 million years ago (mya) until the divergence with Macaque species around 25 mya. At this point, selection acted on ‘CCC' resulting in a nonsynonymous change to ‘CAC’, which altered the encoded amino acid from proline to histidine.

At position 149, the site initially encoded arginine from the shared common ancestor with great apes until the divergence with *Pongo* spp. (Orangutan) approximately 18 mya. These ancestral codon changes at the at the FcγRI binding interface contributed to the formation of an extant inverted CE loop structure in *homo sapiens*. To further understand the functional implications of these changes, we investigated how ancestral sequence changes at FcγRI H^148^/W^149^ sites influence molecular dynamics of IgG binding with FcγRI.

### Modeling of ancestral and alanine mutants at position 148 and 149 reveals their important roles in receptor–Ligand interactions

FcγRI codon substitution models were successfully produced for molecular dynamic studies (Fig. 2). Specifically, H^148^P, W^149^R & H^148^P/W^149^R ancestral FcγRI models were generated. In addition, alanine single (H^148^A, W^149^A) and double substitutions (H^148^A/W^149^A) were also examined. As there is more than one physical interface and numerous glycan interactions between FcγRI and IgG, we adopted both the molecular mechanical/Generalized Born Molecular Volume (MM/GBMV) and Bennett Acceptance Ratio (BAR) [27] methods to determine relative free energy instead of absolute free energy potentials due to the nature of entropy often exhibiting large fluctuations within MD trajectories [28].

Using the MM/GBMV model, we measured the thermodynamic properties of this reaction in three outputs (ligand, receptor, and complex) expressed as the following:

Changes in the gas phase molecular mechanics energy (ΔE_MM_) include both dihedral and angle internal energy (ΔE_el‐st_), while Van der Waals energy (ΔE_vdw_) and solvent accessibility (ΔG_sol_) were also considered in these simulations, with nonpolar sums arising from generalized‐Born and polar capture from GBMV. Entropic vibrational modes were overlooked due to the relative nature of our aim. Entropic vibrational modes were overlooked due to the relative nature of our aim, we performed MD simulations on all substitution models (*n* = 6) and confirmed the importance of the FcγRI H^148^/W^149^ binding to IgG Cγ2‐B chain.

### Binding free energy profiles of FcγRI variants from MD simulations

**Fig. 3:**
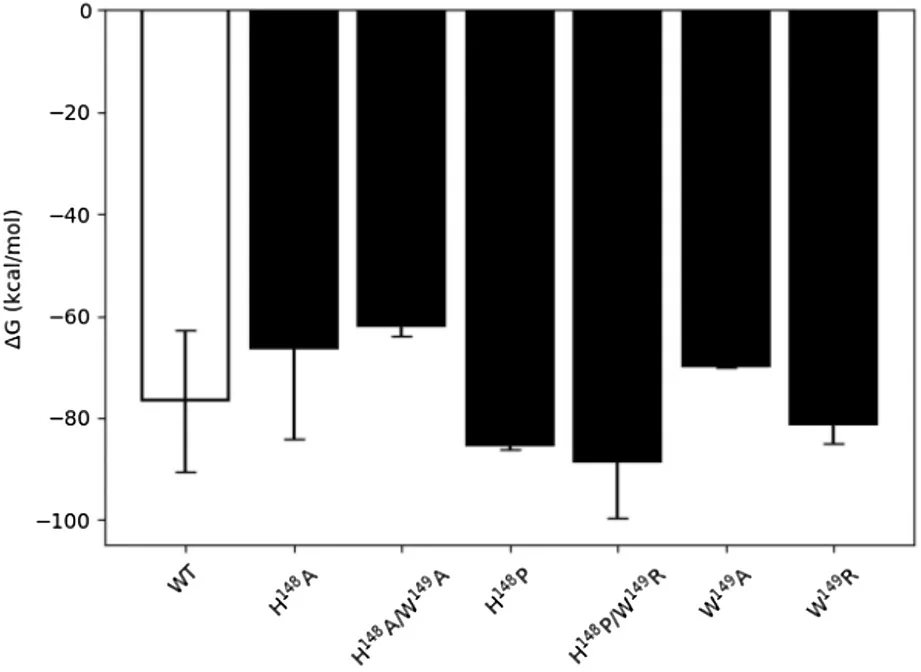
Calculated binding free energies (ΔG) from 100‐ns molecular dynamics simulations (replicate = 1) for wild‐type (WT) and substitution variants. With error bars indicating standard deviations.

Our data indicate all FcγRI variants maintained strong thermodynamically favorable binding to IgG with the wild‐type (WT) exhibiting an average ΔG of −76.67 ± 13.93 kcal·mol^−1^. Among the double mutants, H^148^PW^149^R achieved greatest stability (−88.42 ± 11.33 kcal·mol^−1^), whereas H^148^AW^149^A produced the most destabilizing effect (−61.78 ± 2.14 kcal·mol^−1^). The ancestral single substitutions enhanced stability, with H^148^P averaging a ΔG of −85.20 ± 0.97 kcal·mol^−1^ and W^149^R averaging at −81.23 ± 3.71 kcal·mol^−1^. In contrast, single alanine substitutions reduced binding affinity, with H^148^A at −66.31 ± 17.77 kcal·mol^−1^ and W^149^A at −69.88 ± 0.33 kcal·mol^−1^ (Fig. 3). In summary, ancestral substitutions tend to stabilize FcγRI–IgG interactions relative to WT, while alanine substitutions are destabilizing (ANOVA, *P* = 0.0345). However, subsequent Dunnett's post‐hoc comparisons with WT did not reach significance, indicating that while there is a general trend, pairwise differences should be interpreted with caution (Table 6).

**Table 6:** Summary table of mean binding free energy (ΔG) and standard deviation (SD) for each variant.

Mutant	Mean (kcal·mol^−1^)	SD (kcal·mol^−1^)
WT	−76.67	13.93
H^148^A	−66.31	17.77
H^148^A/W^149^A	−61.78	2.14
H^148^P	−85.2	0.97
H^148^P/W^149^R	−88.42	11.33
W^149^A	−69.88	0.33
W^149^R	−81.23	3.71

### Radius of gyration

The radius of gyration (Rg) is a fundamental structural parameter that characterizes the spatial distribution of atomic mass with respect to a superimposed molecular center, quantifying changes in the complex's compactness across the trajectory. Here, Rg was calculated along two principal axes of inertia for both the receptor (R) and ligand (L), enabling detection of anisotropic conformational changes in the FcγRI–IgG complex arising from substitutions at H^148^ and W^149^.

**Fig. 4:**
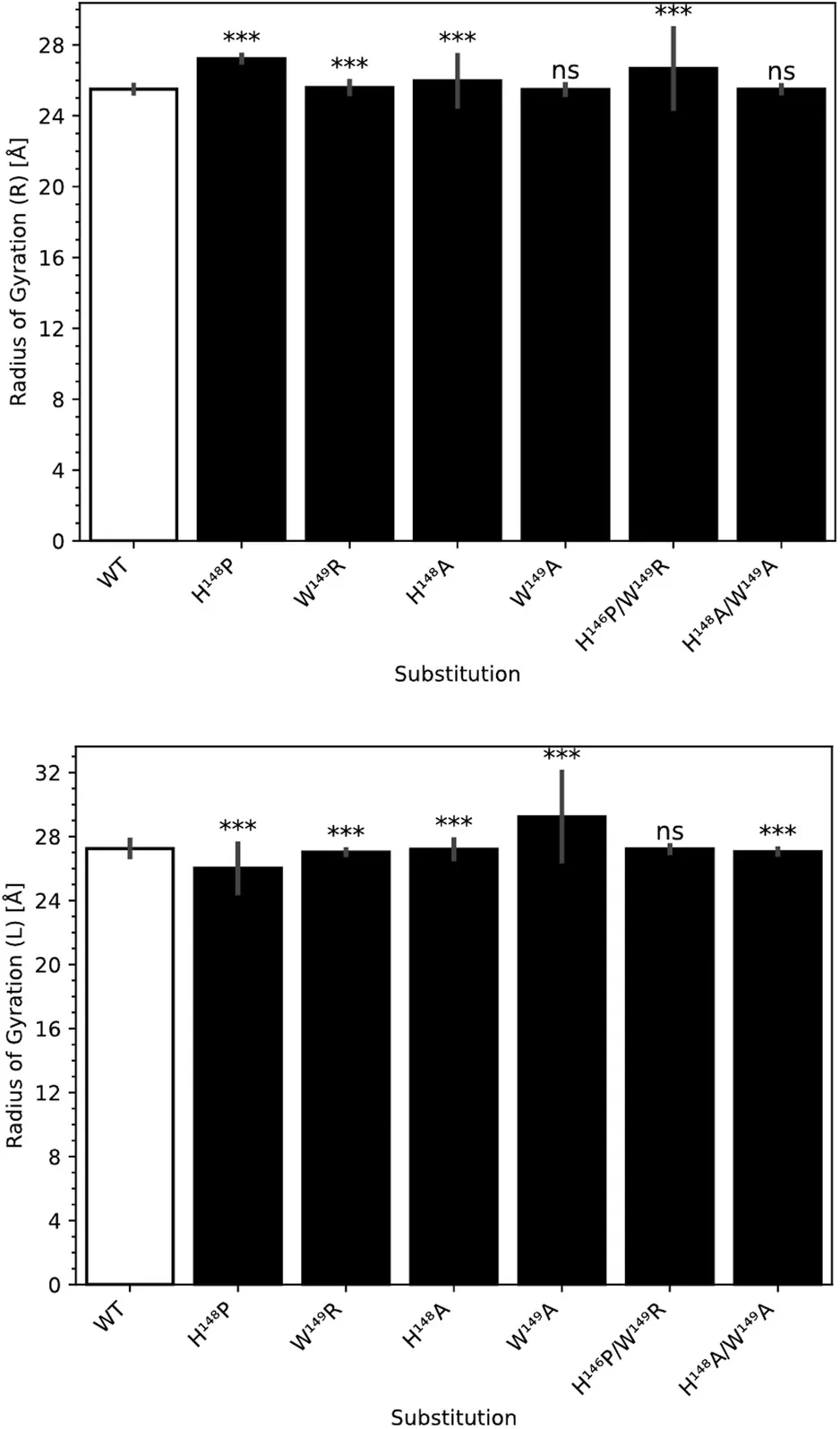
Radius of Gyration of FcγRI Receptor and IgG Ligand. Panels show mean Rg (Å) over 100 ns simulations (replicates = 1): Panel A (R) for the receptor, Panel B (L) for the ligand. Error bars show SD. Significance vs WT: ***P < 0.001, **0.001–0.01, *0.01–0.05, ns ≥ 0.05 (Shapiro–Wilk normality followed by Kruskal–Wallis/Dunn).

WT receptor (R) Rg averaged 25.50 Å, and all ancestral counterparts showed a significant difference compared to WT; H^148^P = 27.23 Å, W^149^R = 25.58 Å, H^148^P/W^149^R = 26.66 Å, demonstrating a *P* = <0.0001 for Shapiro–Wilk normality test. In contrast, the alanine substitutions reveal heterogeneous effects. H^148^A = 25.49 Å (*P* = <0.001), while W^149^A = 24.99 Å (*P* = 0.9996) and H^148^A/W^149^A = 25.5 Å (*P* = 0.9899) from WT (Fig. 4A) were not significant.

Ligand (L) compactness when in receptor–ligand complexes differed significantly from WT across substitutions, except for the double ancestral mutant. In WT simulations, IgG exhibited a mean Rg of 27.25 Å. Single ancestral substitutions significantly altered IgG compactness: H^148^P complex showed a reduced Rg of 25.8 Å (*P* = 0.0001), and the W^149^R complex exhibited a change to 27.02 Å (*P* < 0.0001). In contrast, binding to the double H^148^P/W^149^R ancestral variant resulted in no significant difference relative to WT (28.10 Å; *P* = 0.3447) (Fig. 4B). All alanine substitutions led to significant decreases in ligand compactness compared to WT, with W^149^A showing the largest effect (29.49 Å; *P* = 0.0001), with more moderate increases observed for H^148^A (27.20 Å; *P* = 0.0286) and H^148^A/W^149^A (27.21 Å; *P* = 0.0344) (Fig. 4B).

### Root mean square deviation/fluctuation

Root mean square deviation (RMSD) quantifies the average positional displacement of atoms from a reference structure, providing insight into conformational stability over the simulation. Lower RMSD values generally indicate greater structural stability and, for protein–ligand systems, are often associated with stronger binding. Root mean square fluctuation (RMSF) measures the positional variability of individual residues, offering a localized view of atomic mobility and flexibility. Here, RMSF was examined for FcγRI in complex with IgG, focusing particularly on substitutions at positions 148 and 149. Ligands that remain close to their receptor with a low RMSD have strong binding, while ligands with high RMSD values have weaker binding [29].

**Fig. 5:**
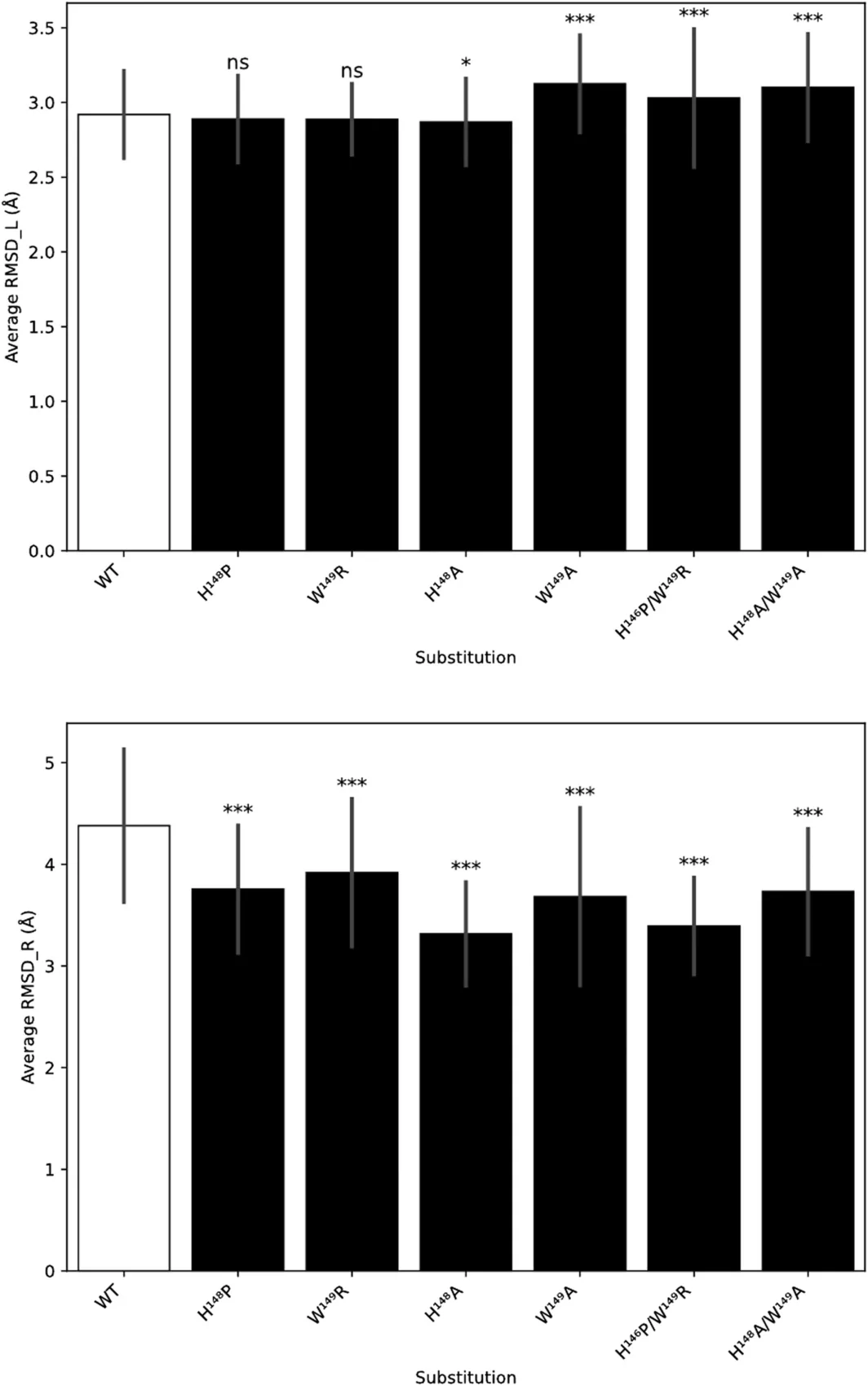
(A) Average RMSD_L (Å) of the ligand bound to WT (white) and mutant (black) receptors, showing mean ± SD, 100 ns simulations (replicates = 1). (B) Average RMSD_R (Å) of the receptor itself for the same variants. Statistical significance vs WT by One‐way ANOVA is indicated above bars (*P < 0.05; **P < 0.01; ***P < 0.001; ns, not significant).

For the IgG ligand (L) in complex with WT, the RMSD averaged 2.92 Å, while the receptor (R) RMSD was 4.38 Å based on standard atomic superpositions. For ancestral variants in complex, H^148^P showed reduced RMSD in both receptor (3.76 Å) and ligand (2.89 Å), as did W^149^R, with ligand (2.89 Å) and receptor (3.92 Å). The double ancestral mutant H^148^P/W^149^R exhibited a modest increase in ligand RMSD (3.03 Å) but a decrease in receptor (3.39 Å). The alanine substitutions showed mixed effects where W^149^A ligand demonstrated (3.19 Å) and receptor (3.72 Å). H^148^A lowered RMSD in both ligand (2.87 Å) and receptor (3.12 Å). The double alanine mutant H^148^A/W^148^A increased RMSD in the ligand (3.09 Å) while decreased in the receptor (3.73 Å) compared to WT data (Fig. 5A,B).

**Fig. 6:**
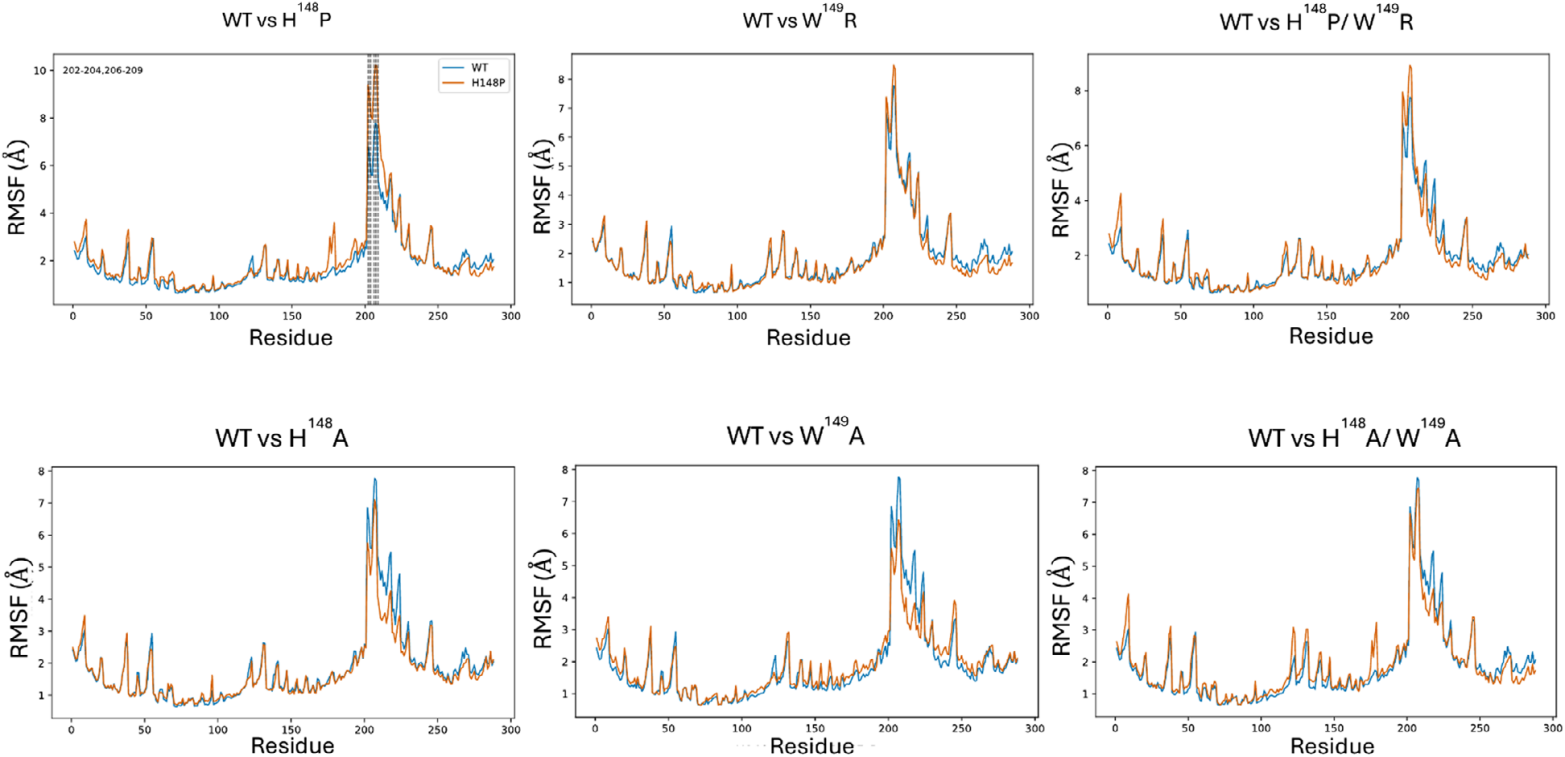
Residue‐level flexibility of FcγRI receptor in WT and mutant complexes. Root‐mean‐square fluctuation (RMSF, Å) of the FcγRI receptor across specific codon positions for WT (blue) and individual receptor mutants (orange). Dashed black vertical lines indicate residues where the mutant deviates from WT by more than two standard deviations of the WT RMSF, with affected residues listed in the top‐left corner of each panel. Data obtained from 100‐ns molecular dynamics simulations.

One‐way ANOVA with *post hoc* analysis revealed all receptor RMSD differences for the substitutions were significantly different from WT (*P* = <0.0001). For the ligand RMSD, both ancestral substitutions demonstrate no significant difference (H^148^P (*P* = 0.1557) and W^149^R (*P* = 0.1087)), respectively. However, in contrast, all other substitutions show significant differences (*P* = <0.0001) (Fig. 5). One‐way ANOVA was also performed on root mean square fluctuations (Fig. 6) and identified no significant difference in codon position fluctuation across the substitutions compared to the WT (*P* = 0.0714) for the receptor. RMSF analysis showed that all FcγRI variants retain overall structural stability, with mutation‐specific increases in flexibility confined to the loop region at residues 200–220. The H^148^P substitutions produce the largest increases in local mobility, suggesting potential functional consequences for Fc‐binding dynamics, whereas alanine substitutions at these sites have minimal structural impact.

### Minimal free energy landscape profiles of WT FcγRI against variants

**Fig. 7:**
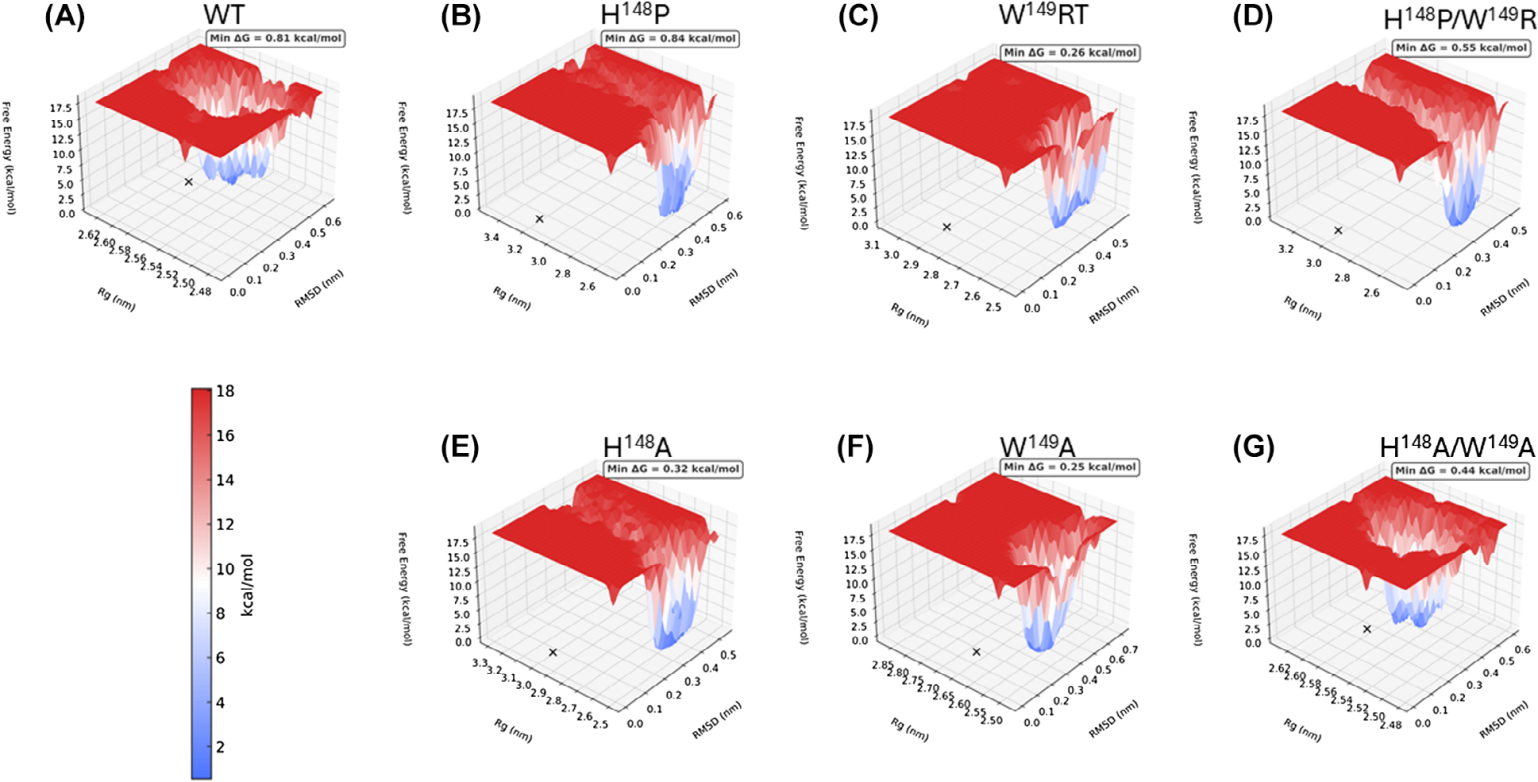
Free‐energy landscapes (FELs) of FcγRI–IgG complexes derived from 100 ns molecular dynamics simulations (replicate = 1), comparing WT to variants. RMSD and radius of gyration (Rg) were used as reaction coordinates, and Gibbs free energy (ΔG, kcal/mol) is color‐mapped from high (red) to low (blue). A = WT, B = H148P, C = W149R, D = H148P/W149R, E = H148A, F = W149A, G = H148A/W149A & H = lowest energy point key where blue = stabilizing and red = destabilizing.

Thermodynamic maps were constructed to demonstrate minima, the most stable, lowest energy form for each variant compared to WT. Our data derived from 100‐ns molecular dynamics simulations show WT minimum ΔG at 0.81 kcal·mol^−1^ (Fig. 7). All the substitutions had a destabilizing increase in energy; substitutions H^148^P showed a ΔG of 0.84 kcal·mol^−1^ with a ΔΔG at −0.03 kcal·mol^−1^ & H^148^A presented a ΔG of 0.32 kcal·mol^−1^ with a ΔΔG at 0.49 Kcal/mol. At position 149, W^149^R showed a ΔG of 0.26 kcal·mol^−1^ with a ΔΔG at 0.55 kcal·mol^−1^, while W^149^A demonstrates ΔG at 0.43 kcal·mol^−1^ with a ΔΔG at 0.26 kcal·mol^−1^. The double mutants had the most destabilizing act with H^148^P/W^149^R ΔG of 0.55 kcal·mol^−1^ with a ΔΔG at 0.26 kcal·mol^−1^ & H^148^A/W^149^A ΔG of 0.44 Kcal·mol^−1^ with a ΔΔG at 0.37 kcal·mol^−1^. The thermodynamic landscape demonstrates a disruption to the native WT stability.

### Hydrogen bond formation

Hydrogen bonds are a key determinant of receptor–ligand stability, and their formation and persistence are directly reflected in the bond energy landscape during molecular dynamics simulations. Over our simulation, we observed stable hydrogen bond formation within the FcγRI–IgG complex, with distinct effects arising from ancestral and alanine substitutions at the H^148^/W^149^ interface.

**Fig. 8:**
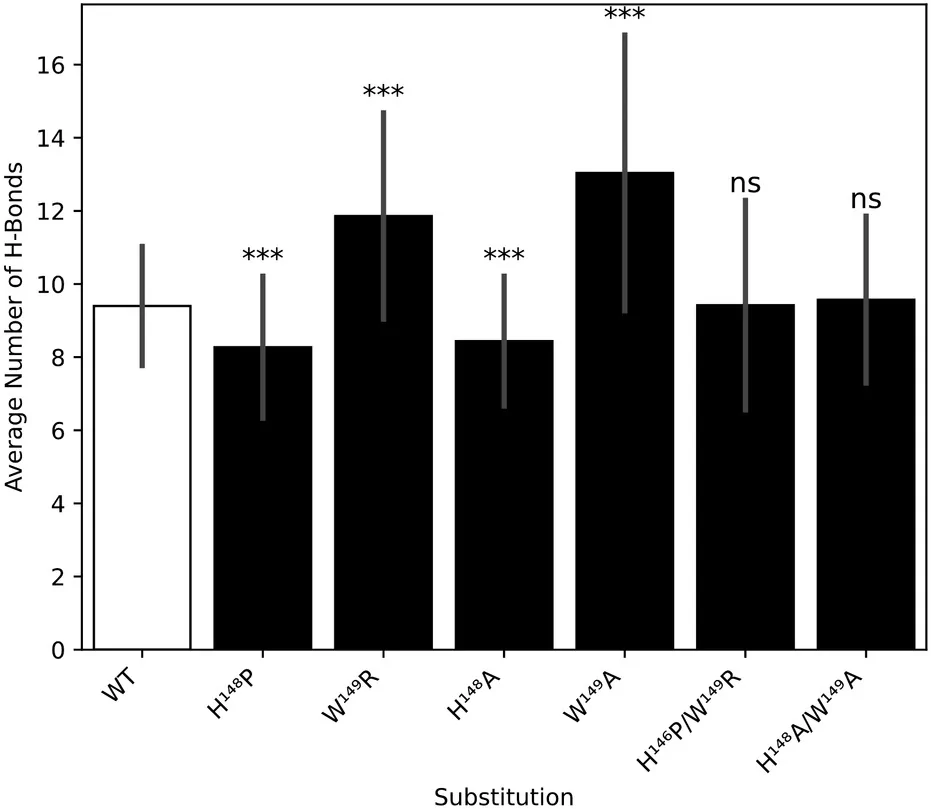
(A) Mean number of hydrogen bonds formed by wild‐type (WT) and mutant receptors during 100 ns molecular dynamics simulations (replicates = 1). Error bars represent standard deviations. Statistical significance compared to WT is indicated: *P < 0.001; ns = not significant. (B) Mean ± standard deviation of hydrogen bond counts for each receptor variant, along with *P*‐values from statistical comparisons to WT (Kruskall–Wallis followed by Dunn *post hoc* comparisons to WT). Variants with *P* < 0.05 are considered significantly different; ns denotes no significant difference.

In the WT complex, hydrogen bond formation averaged 9.4 ± 11. All of the single substitutions were significantly different. This included ancestral single substitutions (H^148^P mean = 8.27, *P* = 0.0001 and W^149^R mean = 11.86, *P* = <0.0001). In addition, the alanine single substitutions (H^148^A = 8.43, *P* = <0.0001 and W^149^R mean = 13.03, *P* = 0.0001) (Fig. 8). The double ancestral substitution, however, showed no significant change in bond formation (mean = 9.42, *P* = 0.9997), and the double alanine substitution was not statistically significant either (H^148^A/W^149^A mean = 9.57, *P* = 0.4497) (Table 7). Changes at position 148 were expected, as H^148^ acts as an acceptor and a donor of electrons, allowing it to interact with multiple partners. Substitutions that disrupt this dual capability (H^148^P, H^148^A) reduced average hydrogen bond counts. W^149^ is structurally significant, and changing this amino acid to arginine (W^149^R) increased the binding interface potential, reducing receptor–ligand interface distance. However, W^149^A saw a significant increase in bond formation, which may be due to the loss of a side chain, which increases the solvent exposure of H^148^ to IgG, and accessibility of alanine's backbone hydrogen. Both double substitutions (H^148^P/W^149^R) and (H^148^A/W^149^A) (Fig. 8) showed no significant changes, which may be due to compensatory local conformational rearrangements, repositioning the receptor to adopt a L^173^ proxy residue contact.

**Table 7:** Mean ± standard deviation of hydrogen bond counts for each receptor variant, along with *P*‐values from statistical comparisons to WT (Kruskall Wallis followed by Dunn *post‐hoc* comparisons to WT). Variants with *P* < 0.05 are considered significantly different; ns denotes no significant difference.

Mutant	Mean ± SD	*P*‐value vs WT
WT	9.40 ± 1.63	–
H^148^A	8.44 ± 1.77	<0.001
H^148^A/W^149^A	9.57 ± 2.28	ns
H^148^P	8.27 ± 1.94	<0.001
H^148^P/W^149^R	9.42 ± 2.87	ns
W^149^A	13.04 ± 3.77	<0.001
W^149^R	11.86 ± 2.82	<0.001

### Destabilization occurs early during simulation

Destabilization time (ns) reflects how long the protein maintains its stable conformation before undergoing a significant structural deviation (RMSD spike). Higher times indicate greater structural stability, while lower times indicate faster destabilization. Destabilization occurred early in the 100‐ns simulation, where the ancestral single substitution W^149^R destabilized first (2.80 ns), followed by the WT (3.80 ns) and then the H^148^P/ W^149^R double ancestral variant (4.10 ns), with all three suggesting quick destabilization. Moderate stability was observed in H^148^A/W^149^A (4.50 ns) and H^148^P (5.3 ns). High stability was demonstrated by variants H^148^A (8.50 ns) and W^149^A (10.6 ns).

## Discussion

### Evolution has driven positive selection at a key FcγRI‐IgG binding interface

Our findings reveal that evolutionary pressures have acted on the FcγRI–IgG binding interface, specifically at residues 148 and 149 as these positions exhibit the strongest signatures of positive selection across multiple selection tests. These H^148^ and W^149^ residues form an inverted C’ loop that constitutes one of two major physical interaction sites with the Fc portion of IgG. The selective events that have shaped this sequence variation, and the potential role of co‐evolution remains a critical question. Prior studies by Machdo *et al*. [16] show pathogen diversity, in particular helminth burden, as a putative driver of functional variation in low‐affinity *FCGR* genes across both mammalian species and human populations. Our data suggest that substitutions at position 148 tend to destabilize the FcγRI–IgG complex as reflected in increased radius of gyration and RMSD/F values. Conversely, changes at position 149 confer an overall stabilizing effect based on the same parameters.

**Fig. 9:**
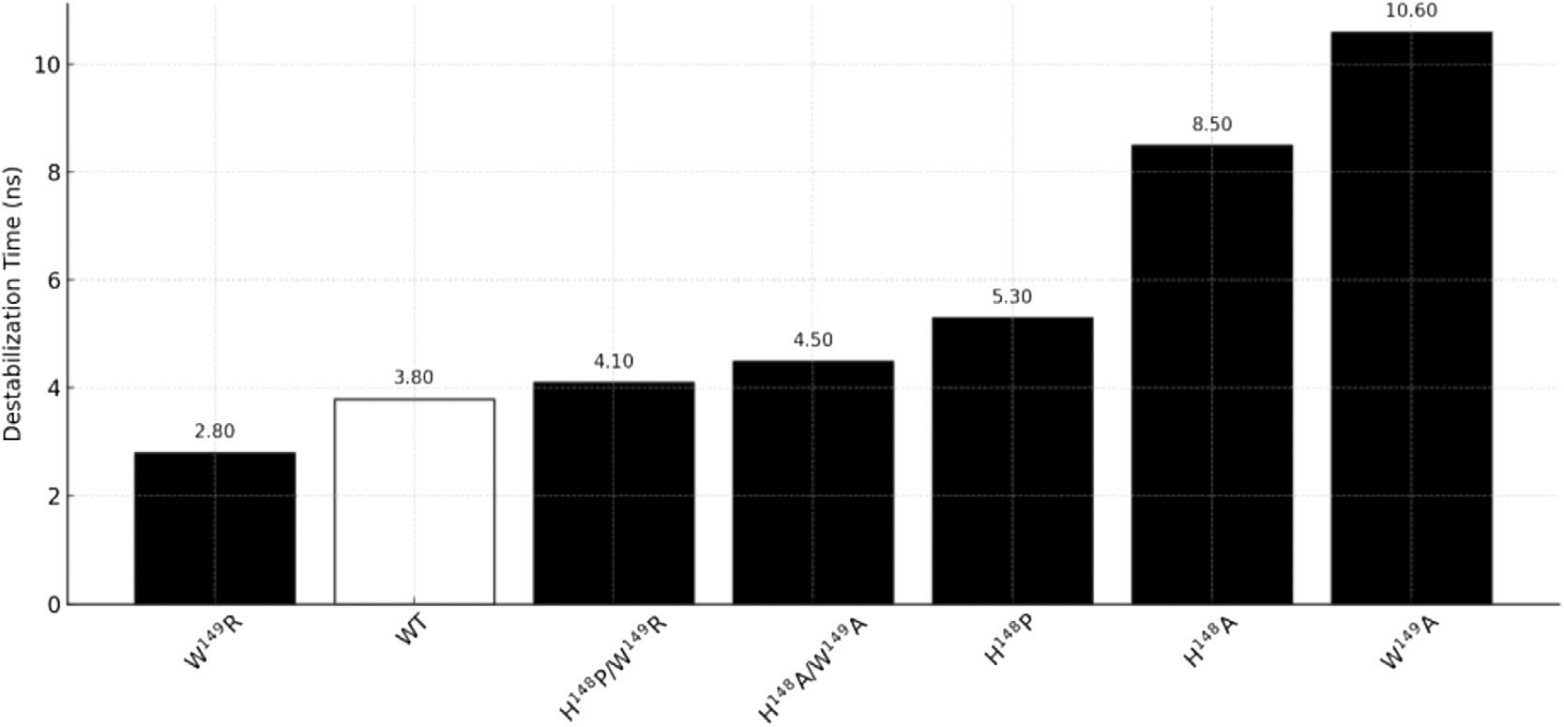
Destabilization time points during the 100 ns simulation. W^149^R was the first simulation to destabilize, followed by the WT, H^148^P/W^149^R, H^148^A/W^149^A, H^148^P, H^148^A, while the W^148^A simulation took longer to destabilize.

However, all substitutions exhibited destabilization at various time points in the simulation (Fig. 9) W^149^R (2.8 ns), potentially due to the loss of hydrophobic packing destabilized quicker than the WT (3.8 ns), while both double ancestral substitutions destabilized less than 1 ns later, H^148^PW^149^R (4.1 ns) and H^148^AW^149^A (4.5 ns) respectively, these substitutions show moderate stabilization/neutral change. H^148^P also demonstrated increase in time to destabilize (5.30 ns) suggesting that while the proline residue provides structural rigidity, it does not compensate for the amphiphilic nature of histidine, acting as an acceptor or donor of hydrogen bonding due to the δ and ε nitrogen's of the imidazole ring. H^148^A (8.50 ns) and W^149^A (10.60 ns) stayed stable the longest, the latter evidencing position 149 as epistatic as single mutations provide the two extreme values of time destabilization (Fig. 9), meaning that position 149 stability is dependent on which amino acid is present at position 148. Position 149 appears to favor interactions where the tryptophan residue can interact with glycan constituents on IgG‐Fc. However, position 149 does not appear to directly bind IgG, but serves a critical structural role. The tryptophan residue at this site stabilizes the inverted C′ motif, optimizing H^148^ exposure for ligand binding at G^235/236^. Our data indicate the evolutionary change to tryptophan at position 149 may have increased the potential for the binding capacity of H^148^ NH donor group by altering backbone conformation to increase accessibility.

Furthermore, FcγRI RMSF data (Fig. 8), flexibility increases at residues 150–165, which are found directly adjacent to the position 148/149 interface under the strongest evidence of selection, and 190–200 correspond to D2 loops βE–βF and βG–βH, located at the additional binding interface, adjacent to the ^173^KHR^175^ motif suggesting interactions. Additional fluctuation at residues 200 maps to the D3 βC–βD loop; this region is implicated in stabilizing receptor orientation relative to the Fc portion of IgG. Increased dynamics here, particularly at L^172^ and P^266^, may weaken hydrogen‐bonding networks due to increased distances for interaction partners such as IgG‐G^235/6^. Consequently, this impacts glycan interactions and nonbonding interactions such as Π‐Π stacking, collectively modulating receptor–ligand affinity and allosteric communication across the extracellular domains. However, this interaction is constrained by the planar geometry of tryptophan's aromatic ring, which enforces a maximum 3 Å distance for effective interaction, which is difficult during molecular fluctuations, and reduces receptor movement. In summary, positive selection has fine‐tuned the FcγRI–IgG interface by modulating both direct ligand contacts and local structural configurations, reflecting an evolutionary trade‐off between stability and flexibility in immune recognition.

Although here we focus on FcγRI, our RMSF data for IgG was also mapped to the FcγRI–IgG1 cocrystal structure (PDB 4W4O) reveals that the residues exhibiting >2 SD deviations align closely with known interface and hinge‐adjacent elements, critical for complex stability. In the IgG‐Fc ligand mutants (panels A–F), elevated flexibility at residues 236–243 corresponds to the lower hinge and CH2 N‐terminal loop, which form key van der Waals contacts with the FcγRI D2 domain. Increased motion in this region suggests a loss of local rigidity that could disrupt optimal receptor recognition. Similarly, residues 297–305, part of the CH2–CH3 junction β‐turn, displayed enhanced fluctuations in several mutants, indicating destabilization of the glycan‐proximal region that normally constrains Fc conformational motion. Conversely, reduced RMSF at residues 428–435 of the CH3 loop implies compensatory rigidification that might limit conformational adaptability during binding, meaning that there appears to be compensatory mechanisms in place for consistent interactions even when structural changes occur.

The flexibility changes observed in the FcγRI–IgG mutant complexes likely translate into altered binding energetics and receptor activation potential. Enhanced mobility within the IgG hinge‐proximal CH2 region (residues 236–243) may reduce the enthalpic stability of receptor engagement by disrupting the precise alignment of Fc loops with the D2 interface, while increased fluctuations at the CH2–CH3 junction (residues 297–305) could affect glycan‐dependent conformational restriction, thereby modulating receptor affinity. On the receptor side, greater flexibility within D2 and D3 loops (residues 150–165, 190–200, and 265–280) is predicted to weaken intermolecular hydrogen bonds and salt bridges that anchor the receptor. These shifts may lower the binding free energy and promote a more transient interaction mode, consistent with dynamic ‘sampling’ of Fc conformations. Functionally, such changes could influence receptor clustering and ITAM‐mediated downstream signaling, potentially attenuating immune effects.

### Tryptophan at position 149 enhances receptor–ligand interactions

Tryptophan possesses a unique electrostatic interaction capability due to its electron‐rich indole system that allows noncovalent π‐cation and anion‐π interactions [30]. This unique interaction occurs in various cationic membrane interactions as demonstrated by [30]. Additionally, tryptophan residues can form hydrogen bonds, creating dipole–dipole and/or dipole‐charge interactions, through hydrophobic C‐H‐π and π‐π stacking interactions. Although the benzine portion of tryptophan lacks a net dipole moment, the carbon sp_2_ atoms have greater electronegativity than adjacent hydrogens, allowing aligned dipolar moments [31] and quadripolar charge distributions, which allow greater interactions with ions such as zinc but also interacting with glycine^235/236^ backbones. Lu *etal* [31] found that in the crystal structure of FcγRI, minimal direct contact occurs between receptor and ligand due to water molecule interactions. Notably, H^174^ is coordinated through zinc ion interactions which makes plausible the rapid evolution of R^149^W.

Furthermore, zinc ions compete with residues of the receptor and ligand, specifically the interaction between the FG loop and N^265^ and the hydroxyl group form hydrogen bonds of a N‐acetylglucosamine that associates with N^297^.

### Fucosylation of FcγRI

Our findings stress the structural significance of positions 148 and 149 to IgG binding, building on the insights of Lu *et al*. [4] who highlighted the second physical binding interface. Adding further complexity, Sakae *et al*. [32] found that fucosylation of IgG also plays a role in cellular responses across the low‐affinity receptors, and this leads to conformational changes. Torsion angle changes and differentiated N‐linkage dynamics influence the tertiary conformation and binding potential of the complex. Specifically, three torsion angles —φ, ψ and ω— show differential flexibility based on fucosylation status. ω remains fixed; however, φ & ψ exhibit flexibility while fucosylated but is reduced in the nonfucosylated state (φ = 77.9 & 78.7° & ψ = 127.5 and 130.8°) (Harbison *et al*. [33]). The N‐Acetyl glucosamine (GlcNAc)(2)β(1–4)–(GlcNAc(1)) glycan moiety remains conformationally rigid, predominantly adopting a single rotameric state (Lu *et al*. [4]). The core fucosylated tetrasaccharide Man β‐(1–4) – GlcNAc β‐(1–4) [α(1–6)‐Fuc] GclNAc adopts a stable ^4^C_1_ chair conformation 87% of the time, yet transitions to the less stable ^1^C_4_ formation the remaining 13%. This conformational flexibility results in a 4.7 kJ·mol^−1^ energy decrease in interaction energy, which negatively affects binding affinity and downstream potential [33].

### pH‐dependent effects of the H^148^ substitution on FcγRI–IgG binding

We investigated whether evolutionary changes at position 148 of FcγRI could influence antibody binding under physiologically relevant pH fluctuations, such as those occurring during acidosis. To assess this, we compared the pK_a_ values of the ancestral proline with the extant human histidine at this site. The ancestral proline had a PKa_1_ = 1.99 and a PKa_2_ = 10.6, with no PKa_3_ due to its lack of an ionizable side chain. In comparison, the positively selected histidine variant displayed a side chain PKa_1_ of 6, close to physiological pH (Edgcomb & Murphy [34]). Histidine may have been favored due to its versatile imidazole ring functioning as an acceptor and donor of electrons via its Nδ^1^ and Nε^2^ atoms. This enhances binding partner adaptability, an ideal property in high‐fluctuating binding environments. In extant FcγRI, H^148^ can bind not only to a glycine G^236/237^ of IgG but also adjacent leucine residues.

The interaction potential of H^148^ is strongly influenced by its protonation status at physiological pH. Histidine is often found with a deprotonated imidazole ring due to π systems of resonance stabilization. Given the two nitrogen positions (Nδ1 and Nε2), an average PKa_3_ value of ~6 is often used as approximate protonation behavior [34]. Applying the Henderson Hasselbach equation (Equation 1) and at a standard physiological pH of 7.4 yields an estimated distribution of 3.83% protonated and 96.17% deprotonated H^148^. This deprotonated state has a high binding affinity for partner glycine residues of IgG where 99.37% is protonated compared with 0.63% deprotonated, making the interaction highly beneficial and energetically advantageous in the FcγRI–IgG interface.

Standard Henderson Hasselbach formula:

(1) $$\mathrm{pH}={\mathrm{PK}}_{a}+{\mathrm{Log}}_{10}{\left.\left(\;\frac{A-}{\mathrm{HA}}\right.\right)}^{n}$$

Equation 1: Henderson Hasselbach equation, PK_a_ is dissociation constant, *A*− = deprotonated form for histidine, HA = protonation of histidine.

Using the Henderson Hasselbach (Equation 1), we evaluated protonation status of H^148^ under conditions mimicking sepsis‐associated acidosis (pH 6.8). At this lower pH, histidine had a decreased protonation fraction (0.14% protonated vs 99.86% deprotonated) correlating with a decrease in binding potential. In contrast, the ancestral proline is predominantly protonated (99.93%) at physiological pH at the secondary amine nitrogen, while the carboxyl group remains deprotonated, steadily becoming protonated as pH becomes acidic, where full protonation occurs at pH 6.8. Notably, G^236/237^ on IgG remains protonated at both pH 7.4 (99.37%) and pH 6.8 (99.84%) further highlighting H^148^ as a residue better suited for sustaining ligand engagement in fluctuating pH environments. However, it is important to note these are theoretical calculations, and future studies would benefit from physical laboratory tests.

Equation 2: A: MM/GBSA Binding Free Energy Equation, B: Molecular Mechanics Energy Decomposition, C: Solvation Free Energy Decomposition.

$$A:\;\Delta G_{\text{bind}}=\Delta H-T\Delta S\;\approx \;\Delta E_{\mathrm{mm}}+\Delta G_{\mathrm{sol}}-T\Delta S$$

$$B:\;\Delta E_{\mathrm{mm}}=\Delta E_{\mathrm{int}}+\Delta E_{\mathrm{el}-\mathrm{st}}+\Delta E_{\mathrm{vdw}}$$

(2) $$C:\;\Delta G_{\mathrm{sol}}=\Delta G_{\mathrm{GB}}+\Delta G_{\mathrm{MV}}$$

MM/GBSA binding free‐energy decomposition. The binding free energy is estimated in equation A. The molecular mechanics energy difference determined in equation B. The sum of the internal energy ($\Delta E_{\mathrm{int}}$), electrostatic energy ($\Delta E_{\mathrm{el}-\mathrm{st}}$), and van der Waals energy ($\Delta E_{\mathrm{vdw}}$). The solvation free‐energy term is determined in equation C which includes polar contribution computed by the Generalized Born model ($\Delta G_{\mathrm{GB}}$) and the non‐polar contribution related to molecular volume or solvent‐accessible surface area ($\Delta G_{\mathrm{MV}}$). Binding free energy; ΔH, enthalpy; T, temperature; ΔS, entropy; $\Delta E_{\mathrm{mm}}$, molecular mechanics energy; $\Delta G_{\mathrm{sol}}$, solvation free energy.

## Conclusion

While prior biophysical studies have largely focused on the FG‐loop and glycan‐mediated interactions, our combined evolutionary and molecular dynamics simulations analyses reveal that the adjacent binding site, with additional glycan‐glycan interactions, warrant closer examination. Our simulations show that substitution changes at position 148 destabilize the FcγRI–IgG complex, but changes at position 149 enhance stability. When both positions are substituted, the greater stability generated at position 149 outweighs the instability generated at position 148, resulting in complexes that have greater stability than the wild‐type. This enhanced stability at position 149 is linked to the formation of additional hydrogen bonds, as tryptophan is a poor hydrogen bond partner compared to histidine at position 148, which can act as both an acceptor and donor of protons. However, histidine's protonation state is pH dependent, and the interaction potential reduces under acidic pH, such as those found in hypoxic tissue microenvironments or during sepsis [35]. These studies identify signatures of positive selection at a crucial interface for ligand binding, and this could be due to host‐pathogen co‐evolution. Furthermore, evolutionary studies of IgG are warranted, although challenging to implement due to the variable nature of immunoglobulin isotypes. Molecular modeling and dynamics simulations identified differentiated conformational states occurring over the 100‐ns simulation. The changes differ between ancestral substitutions compared to the alanine variants, where all the ancestral substitutions exhibit an overall increase in stability, in contrast to the alanine substitutions. This may have occurred to allow for greater flexibility in the receptor to associate with IgG. These changes suggest that when rigid at the 148/149 location, the receptor has greater potential for interaction and is less sensitive to pH changes. While extended simulations and/or multiple independent replicas would enhance statistical confidence and capture potential rare conformational events, our focused analysis provides meaningful insights into the conformational landscape of the FcγRI–IgG binding interface, which sets the foundation for future biophysical and cellular studies aimed at experimental confirmation.

## Conflict of interest

The authors declare no conflict of interest.

## Author contributions

DY and LRM conceptualized the study. FG performed preliminary evolutionary experimental work and data collection. DY and OAG conducted computational analyses and contributed to data interpretation. MC provided theoretical expertise and assisted with data interpretation. MG provided evolutionary expertise. DY and LRM drafted the manuscript with contributions from all authors. All authors reviewed and approved the final version of the manuscript.

## Supporting information


**Fig. S1.** Root mean square deviation for WT vs ancestral substitutions.
**Fig. S2.** Root Mean Square Deviation for WT vs alanine substitutions.
**Fig. S3.** Radius of gyration data for WT vs ancestral substitutions.
**Fig. S4.** Radius of gyration for WT vs alanine substitutions.

## Data Availability

The sequence data that support the findings of this study are openly available in Ensembl (https://www.ensembl.org/index.html). The structural model used (PDB 4W4O) in this study was obtained from the Protein Data Bank (https://www.rcsb.org/structure/4W4O). In addition, the data obtained from the molecular dynamics simulations are available from the University of Northampton DOI: 10.24339/19e25b73‐0f86‐40ea‐bb2c‐08689017389a.
