# Supplementary material for: Molecular dynamics simulations of positively selected codons in FcγRI reveal novel biochemical binding properties
Source: FEBS Open Bio. 2026 May 1;16(9):1708–25. doi: 10.1002/2211-5463.70247 (PMC13398953; doi:10.1002/2211-5463.70247)
Supplement: Supplementary file 1 [file FEB4-16-1708-s001.docx]

Supplementary figures

Supplementary figure 1 – Root Mean Square Deviation for WT vs ancestral substitutions

*Supp. Data 1: RMSD fluctuations for the ancestral substitutions over 100 ns, substitutions in red compared to the WT fluctuations in black.*

Supplementary figure 2 – Root Mean Square Deviation for WT vs alanine substitutions

*Supp. Data 2: RMSD fluctuations for the alanine substitutions over 100 ns, with substitutions in red compared to the WT fluctuations in black.*

Supplementary figure 3 – Radius of gyration data for WT vs ancestral substitutions

*Supp. Data 3: ligand (L) and receptor (R) radial gyration of the single ancestral substitutions and double ancestral substitutions*

Supplementary figure 4 – Radius of gyration for WT vs alanine substitutions

*Supp. Data 4: ligand (L) and receptor (R) radial gyration of the single ancestral substitutions and double ancestral substitutions*
