# Supplementary material for: Molecular dynamics simulations of positively selected codons in FcγRI reveal novel biochemical binding properties
Source: FEBS Open Bio. 2026 May 1:10.1002/2211-5463.70247. Online ahead of print. doi: 10.1002/2211-5463.70247 (PMC13398953; doi:10.1002/2211-5463.70247)
Supplement: Supplementary file 1 — Fig. S1. Root mean square deviation for WT vs ancestral substitutions. Fig. S2. Root Mean Square Deviation for WT vs alanine substitutions. Fig. S3. Radius of gyration data for WT vs ancestral substitutions. Fig. S4. Radius of gyration for WT vs alanine substitutions. [file FEB4-9999-0-s001.docx]

Supplementary figures

Supplementary figure 1 – Root Mean Square Deviation for WT vs ancestral substitutions

*Supp. Data 1: RMSD fluctuations for the ancestral substitutions over 100 ns, substitutions in red compared to the WT fluctuations in black.*

Supplementary figure 2 – Root Mean Square Deviation for WT vs alanine substitutions

*Supp. Data 2: RMSD fluctuations for the alanine substitutions over 100 ns, with substitutions in red compared to the WT fluctuations in black.*

Supplementary figure 3 – Radius of gyration data for WT vs ancestral substitutions

*Supp. Data 3: ligand (L) and receptor (R) radial gyration of the single ancestral substitutions and double ancestral substitutions*

Supplementary figure 4 – Radius of gyration for WT vs alanine substitutions

*Supp. Data 4: ligand (L) and receptor (R) radial gyration of the single ancestral substitutions and double ancestral substitutions*
